# Liver mobilization during Kasai portoenterostomy: retrospective multicentre analysis

**DOI:** 10.1093/bjsopen/zrag064

**Published:** 2026-06-08

**Authors:** Marie Uecker, Maximilian Holweg, Maria Hukkinen, Lucas Moratilla-Lapeña, Kristine Dräger, Alexander Domasch, Katja Nickel, Sören Wiesner, Cornelius Jakob van Beekum, Jörg Fuchs, Uta Herden, Francisco Hernández, Mikko Pakarinen, Riccardo Superina, Joachim F Kuebler, Christoph Slavetinsky, Omid Madadi-Sanjani

**Affiliations:** Department of Pediatric Surgery, Hannover Medical School, Hannover, Germany; Department of Pediatric Surgery, University Medical Center Hamburg-Eppendorf, Hamburg, Germany; Department of Pediatric Surgery and Urology, University Children’s Hospital Tübingen, Tübingen, Germany; Section of Pediatric Surgery, New Children’s Hospital, University of Helsinki, Helsinki, Finland; Department of Paediatric Surgery, La Paz Children’s University Hospital, Madrid, Spain; Department of Pediatric Surgery, University Medical Center Hamburg-Eppendorf, Hamburg, Germany; Department of Pediatric Surgery, Hannover Medical School, Hannover, Germany; Clinic of Anesthesiology and Intensive Care Medicine, Hannover Medical School, Hannover, Germany; Institute of Biostatistics, Hannover Medical School, Hannover, Germany; Department of General, Visceral, & Transplant Surgery, Transplant Center Hannover, Hannover Medical School, Hannover, Germany; Department of Pediatric Surgery and Urology, University Children’s Hospital Tübingen, Tübingen, Germany; Department of Visceral Transplantation, University Medical Center Hamburg-Eppendorf, Hamburg, Germany; Department of Paediatric Surgery, La Paz Children’s University Hospital, Madrid, Spain; Section of Pediatric Surgery, New Children’s Hospital, University of Helsinki, Helsinki, Finland; Division of Transplant and Advanced Hepatobiliary Surgery, Ann and Robert H. Lurie Children’s Hospital of Chicago, Chicago, Illinois, USA; Department of Pediatric Surgery and Urology, Hospital Bremen-Mitte, Bremen, Germany; Department of Pediatric Surgery and Urology, University Children’s Hospital Tübingen, Tübingen, Germany; Department of Pediatric Surgery, University Medical Center Hamburg-Eppendorf, Hamburg, Germany; Department of Visceral Transplantation, University Medical Center Hamburg-Eppendorf, Hamburg, Germany

**Keywords:** biliary atresia, native liver survival, pediatric liver transplantation, intraoperative management

## Abstract

**Background:**

Liver mobilization (LM) during Kasai portoenterostomy (KPE) is used by some surgeons to improve exposure but remains controversial due to anaesthetic challenges and concerns about increased adhesions complicating liver transplantation (LT). This study evaluated the impact of LM on intraoperative, postoperative, and long-term outcomes in biliary atresia.

**Methods:**

A multicentre case-control study involving high-volume biliary atresia centres in Europe and North America was conducted. Preoperative, intraoperative, and postoperative clinical and laboratory data were retrospectively collected and comparisons were made between groups with and without LM. Long-term native liver survival was assessed by Kaplan–Meier analysis with log-rank testing.

**Results:**

In all, 204 patients were included in the study: 142 in the LM group and 62 in the No LM group. During KPE, the LM group received catecholamines more frequently (100 (100%) *versus* 13 (46%) patients; *P* < 0.001), required more intravenous fluids (116.2 *versus* 79.15 ml/kg; *P* < 0.014), and had higher transfusion rates (18.91 *versus* 7.59 ml/kg; *P* < 0.001) than the No LM group. After KPE, transaminases were elevated in both groups, but significantly higher in the LM group (aspartate aminotransferase, 1166 *versus* 374.6 U/l; *P* < 0.001; alanine aminotransferase 456.8 *versus* 206.7 U/l; *P* < 0.001). These values normalized within 7 days after KPE. During subsequent LT, patients who had undergone LM experienced significantly shorter operative times (295.3 *versus* 509.8 minutes; *P* < 0.001) and required lower blood transfusion volumes (55.26 *versus* 95.81 ml/kg; *P* < 0.001). Of note, patients who underwent LM showed significantly prolonged native liver survival (*P* < 0.024).

**Conclusions:**

LM during KPE leads to increased intraoperative haemodynamic compromise requiring more intensive anaesthetic management and causes transient postoperative hepatic stress. LM does not appear to negatively affect LT and is associated with improved long-term native liver survival. These findings suggest potential benefits of LM during KPE, warranting further investigation in prospective studies.

## Teaser

This multicentre retrospective study compared outcomes of Kasai portoenterostomy performed with *versus* without liver mobilization (LM) across major European and North American centres. LM was associated with greater intraoperative haemodynamic instability and transient postoperative hepatic stress, but significantly improved long-term native liver survival without adversely affecting later liver transplantation. These findings suggest that, despite short-term physiological burden, LM may confer a meaningful long-term advantage in biliary atresia.

## Introduction

Biliary atresia (BA) is a progressive obstructive cholangiopathy of infancy with unclear aetiology, characterized by destruction of extrahepatic bile ducts, progressive cholestasis, and ultimately liver failure. The primary treatment for BA is Kasai portoenterostomy (KPE), during which the remnant extrahepatic bile ducts are resected and a Roux-en-Y loop is placed on the porta hepatis to restore biliary drainage. Success rates of KPE, defined by clearance of jaundice and native liver survival, vary from 20% to 70%^1–3^, but most patients ultimately require liver transplantation (LT)^4^.

**Fig. 1:**
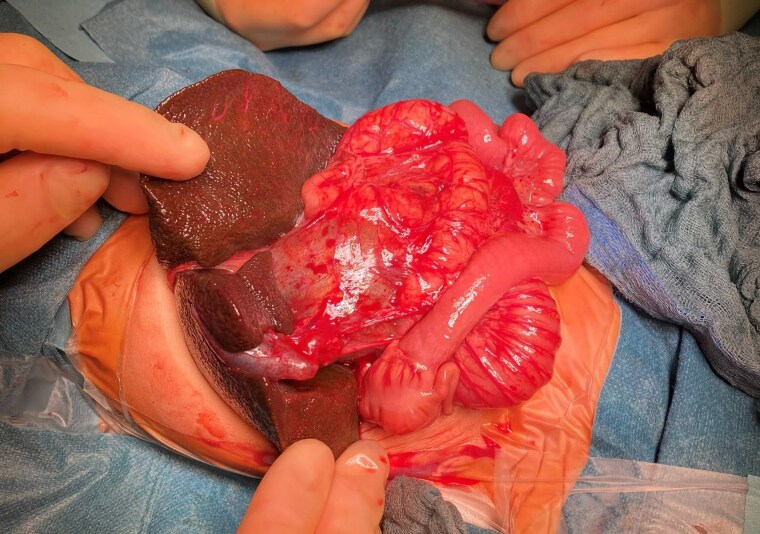
Fully mobilized liver in a patient with cystic biliary atresia

The most critical step during KPE is the meticulous resection of the remnant biliary structures embedded in fibrotic scar tissue on the portal plate, requiring adequate visualization of the porta hepatis and local vascular anatomy. The widely accepted surgical approach includes a degree of mobilization and partial or total exteriorization of the liver for improved exposure of this region (*Fig. 1*)^5,6^.

Despite facilitating portal plate dissection, liver mobilization (LM) has distinct disadvantages. Haemodynamic instability, caused by kinking of the inferior vena cava and subsequent reduction in venous return, often necessitates advanced anaesthetic management^7,8^. An increase in liver adhesions has been reported for patients undergoing LM during KPE, complicating hepatectomy during LT^9^. Studies in adult patients suggest higher transfusion and bleeding rates with extensive LM in oncological hepatobiliary procedures, yet these findings are not directly transferable to paediatric surgery^10,11^.

A recent Chinese single-centre retrospective study^12^ comparing KPE with and without LM, found that the LM group experienced greater intraoperative blood pressure fluctuation, longer postoperative recovery, higher drainage output, and increased length of hospital stay. Short- and long-term outcomes were similar, leading the authors to suggest a no-LM approach may be preferable^12^. Park *et al*.^13^ reported comparable findings in their single-centre experience and the authors concluded that the no-LM KPE may be preferred. However, that study did not assess intraoperative or transplant-related outcomes^13^. Comprehensive data on the effects of LM during KPE remain limited.

The aim of this multicentre study was to analyse the perioperative effects and postoperative outcomes of BA patients with and without LM during KPE.

## Methods

### Data collection

The study was performed in accordance with the Declaration of Helsinki and the study protocol was approved by the institutional ethics committee of the Medical School Hannover (Germany) (No. 10146_BO_SK_2022). Anonymized data were shared between centres after obtaining appropriate data sharing agreements.

High-volume BA centres, defined as institutions performing a minimum of five cases per year and/or specialized centres in countries with centralization of BA care, were selected for participation in the study, with all European centres being members of the ERN Rare-Liver network^14^. Data were collected from three paediatric surgery departments that mobilize the liver during KPE and three departments that routinely perform KPE without LM. Each centre adhered to a consistent surgical approach throughout the entire study period and did not change its operative technique with respect to LM. LM was defined as the complete or partial exteriorization of the liver from the abdominal cavity during KPE, with or without mobilization of the hepatic ligaments. In contrast, centres not performing LM maintained the liver entirely within the abdominal cavity, exposing the hepatoduodenal ligament and porta hepatis exclusively through manual traction without organ exteriorization.

Data were retrospectively collected for patients who underwent both KPE and LT at the respective participating centre with data available for a minimum 1-year follow-up after KPE. Patients with syndromal or cystic variants of BA and infants with co-morbidities affecting haemodynamics or surgical approach (for example, cardiac-associated BA, previous abdominal surgery) were excluded. Data were collected from the preoperative, intraoperative (anaesthetic and surgical), and postoperative periods, including transplant surgery. Preoperative data were collected from the time point closest to surgery, up to a maximum of 7 days before KPE. Postoperative laboratory data were collected from postoperative day (POD) 1 and POD7, with a permissible window of ±2 days. Postoperative complications were assessed using the Clavien–Madadi classification^15^. Prolonged intubation was defined as mechanical ventilation exceeding 24 hours after KPE. Central line-associated bloodstream infection was defined as a positive blood culture obtained from a catheter tip submitted for microbiological analysis. Cholangitis was defined according to the Biliary Atresia and Related Diseases (BARD) criteria^16^ where sufficient documentation was available; in earlier cases, clinical documentation alone was used and a standardized definition could not be applied uniformly. The clinical outcome was assessed at 6 months after surgery and at the longest available follow-up, categorized as jaundice free, persistent jaundice, or LT. For the 6-month outcome comparison, persistent jaundice and LT were combined into a single non-jaundice-free category as a composite indicator of unsuccessful early biliary drainage, consistent with established outcome reporting for BA.

### Statistical analysis

Data were analysed using GraphPad Prism^®^ Version 10.4.1 (GraphPad Software, LLC, San Diego, CA, USA). Data are presented as the mean and standard deviation (s.d.). Continuous variables were analysed using unpaired *t* tests, with non-parametric sensitivity analyses performed where appropriate, given sample size and distributional considerations. Categorical variables were compared using Fisher’s exact test or the χ^2^ tests. Long-term survival with native liver was assessed using Kaplan–Meier estimation with log-rank testing, and results are reported with their 95% confidence interval (c.i.). The level of significance was set at *P* < 0.05, with interpretation of *P* values being exploratory.

## Results

In all, 204 BA patients from six centres were included in the study. The LM group comprised 142 patients from three centres, whereas the No LM group comprised 62 patients from three separate centres. The time period of data collection ranged from 3 to 17 years (maximum time span January 2006–February 2023) depending on caseloads of individual centres.

There was no significant difference in age between the LM and No LM groups at the time of surgery (*P* = 0.43). Patients in the No LM group had significantly lower body weight (*P* = 0.017) and lower bilirubin levels (*P* = 0.034), and higher levels of γ-glutamyl transpeptidase (GGT) and albumin (*P* = 0.001 and *P* = 0.018 respectively; *Table 1*). Preoperative ultrasound results were available for 198 patients and showed antegrade portal vein flow in all cases.

**Table 1:** Preoperative data

	LM group		No LM group		P*
	Mean(s.d.)	*n*	Mean(s.d.)	*n*	
Age at KPE (days)	60.15(25.88)	142	56.97(27.73)	62	0.43
Body weight (g)	4483(800)	100	4160(879)	62	0.017
AST (U/l)	232.4(162.7)	135	231.9(147.5)	60	0.983
ALT (U/l)	161.0(142.6)	138	152.2(101.2)	60	0.665
GGT (U/l)	504.7(385.6)	139	728.3(534.7)	60	0.001
Albumin (mg/dl)	34.38(6.27)	81	37.02(5.53)	47	0.018
**Bilirubin (mg/dl)**					
Total	9.47(3.35)	140	8.17(2.69)	50	0.014
Direct	7.11(2.54)	141	6.19(2.02)	54	0.018

LM, liver mobilization; s.d., standard deviation; KPE, Kasai portoenterostomy; AST, aspartate aminotransferase; ALT, alanine aminotransferase; GGT, γ-glutamyl transpeptidase. *Unpaired *t*-test.

### Intraoperative data

Most patients (97, 48%) were provided with both central venous and arterial vascular access (LM, 55; No LM, 42), whereas 33 patients were given only a central venous catheter (LM, 32; No LM, 1). Eight patients received an arterial and peripheral line (LM, 4; No LM, 4) and 24 patients underwent surgery with peripheral access only (LM, 9; No LM, 15).

Surgical duration did not differ significantly between the two groups (*P* = 0.147; *Table 2*). Patients who underwent LM were more frequently administered catecholamines (*P* < 0.001) and received significantly greater volumes of intravenous fluids and blood transfusions, normalized to body weight (*P* = 0.013 and *P* < 0.001, respectively; *Table 2*). By the end of surgery, lactate levels were significantly higher in the LM group (*P* = 0.002; *Table 2*).

**Table 2:** Intraoperative data

	LM group		No LM group		P*
	Mean(s.d.)	*n*	Mean(s.d.)	*n*	
Surgery duration (min)	186.5(47.2)	100	198.9(46.1)	44	0.147
Intravenous fluids† (ml/kg)	116.2(77.3)	100	79.15(70.40)	35	0.013
Blood transfusion (ml/kg)	18.91(17.46)	100	7.6(12.2)	62	< 0.001
Administration of catecholamines, *n* (%)	100 of 100 (100%)		13 of 28 (46%)		< 0.001
**Lactate (mmol/l)**					
Before surgery	1.2(0.7)	100	1.04(0.35)	18	0.74
At the end of surgery	2.4(1.0)	100	1.58(0.48)	21	0.001
**pH**					
Before surgery	7.33(0.06)	100	7.38(0.06)	22	< 0.001
At the end of surgery	7.31(0.06)	100	7.32(0.06)	22	0.461
**Base excess**					
Before surgery	−2.6(2.3)	100	−0.3(2.6)	21	< 0.001
At the end of surgery	−3.8(2.8)	100	−3.3(3.6)	20	0.702
Abdominal drain, *n* (%)	93 of 142 (65.5%)		55 of 62 (89%)		

^†^Excluding transfusion and medication. LM, liver mobilization; s.d., standard deviation; min, minutes. *Unpaired *t*-test.

### Postoperative data

In patients who underwent LM, abdominal drains were removed later than in the No LM group (*P* = 0.033; *Table 3*). Diuretic therapy was more frequently administered in the LM group (*P* = 0.001). Both groups showed an increase in aminotransferases compared with preoperative levels; this elevation was significantly more pronounced in the LM group (*P* < 0.0001; *Table 3*). By POD7, values had declined to below preoperative levels in both groups (*Table 3*). Similar to preoperative findings, GGT levels remained consistently higher in the No LM group throughout the postoperative course (*P* < 0.001; *Table 3*). Postoperative Doppler ultrasound confirmed antegrade portal vein flow in all patients, except one in the LM group (185).

**Table 3:** Post-Kasai portoenterostomy data

	LM group		No LM group		P*
	Mean(s.d.)	*n*	Mean(s.d.)	*n*	
Time to drain removal (days)	11.56(10.36)	62	8.44(2.5)	54	0.033
Use of diuretics, *n* (%)	76 of 100 (76%)		4 of 28 (14%)		0.001
**POD 1**					
AST (U/l)	1166(1325)	111	374.6(240.2)	53	< 0.001
ALT (U/l)	456.8(415.4)	124	206.7(122.9)	52	< 0.001
GGT (U/l)	282.4(213.6)	102	579.1(402.2)	42	< 0.001
Bilirubin (mg/dl)					
Total	8.46(2.87 )	132	6.36(2.05)	51	< 0.001
Direct	6.55(2.46)	91	5.13(1.58)	19	0.037
Albumin (mg/dl)	24.88(3.76)	98	26.40(5.08)	19	0.133
**POD 7**					
AST (U/l)	141.1(99.9)	138	163.4(82.0)	59	0.013
ALT (U/l)	117.5(80.6)	139	137.5(71.6)	60	0.099
GGT (U/l)	534.1(400.4)	134	1110(810)	54	< 0.001
Bilirubin (mg/dl)					
Total	6.65(2.69)	141	7.01(3.63)	60	0.441
Direct	5.5(2.4)	137	6.05(3.04)	43	0.214
Albumin (mg/dl)	27.82(6.32)	77	30.85(7.75)	28	0.044

Serum concentrations of AST, ALT, GGT, bilirubin, and albumin are reported. LM, liver mobilization; s.d., standard deviation; POD, postoperative day; AST, aspartate aminotransferase; ALT, alanine aminotransferase; GGT, γ-glutamyl transpeptidase. *Unpaired *t*-test.

In all, 42 patients in the LM group and 7 in the No LM group experienced at least one complication (*Table 4*). Cholangitis was the most frequently recorded complication, occurring significantly more often in the LM group (*P* = 0.002). Among patients requiring revision surgery, the most common indications for reoperation included leakage of the Roux-en-Y anastomosis (LM, 3; No LM, 1), or bile leakage of the portoenterostomy (LM, 2; No LM, 1), followed by intra-abdominal bleeding with abdominal compartment syndrome (LM, 2). In the LM group, one patient underwent drainage of an abdominal fluid collection, and one patient required relaparotomy due to a bowel perforation distal to the Roux-en-Y anastomosis.

**Table 4:** Postoperative complications by surgical group

Complication	LM group (*n* = 142)	No LM group (*n* = 62)	P*
Any complication	42 (30%)	7 (11%)	0.004
Cholangitis	35 (25%)	4 (7%)	0.002
Pulmonary embolism	1 (1%)	0	> 0.99
Central line infection	1 (1%)	0	> 0.99
Relaparotomy	9 (6%)	2 (3%)	0.514
Prolonged intubation	0	1 (2%)	> 0.99

Values are *n* (%). LM, liver mobilization. *Unpaired *t*-test.

### Outcomes after KPE

The mean(s.d.) follow-up duration was 68.36(48.77) months in the LM group and 55.67(22.85) months in the No LM group.

**Fig. 2:**
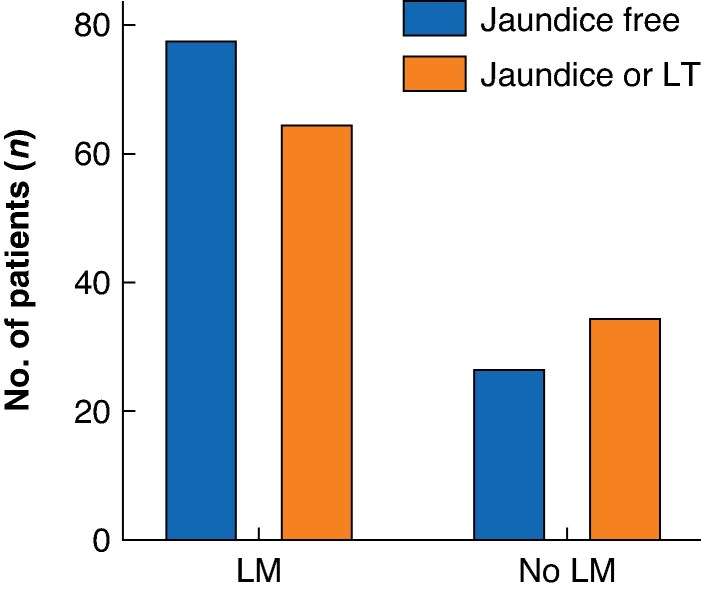
Clinical outcomes 6 months after KPE comparing jaundice-free and jaundice or LT groups with or without LM There were 142 patients in the LM group: 78 jaundice free and 64 with jaundice or LT. There were 62 patients in the No LM group: 27 jaundice free and 35 with jaundice or LT. Differences between LM- and no LM-Group were analysed using Fisher’s exact test (*P* = 0.171). KPE, Kasai portoenterostomy; LT, liver transplantation; LM, liver mobilization.

**Fig. 3:**
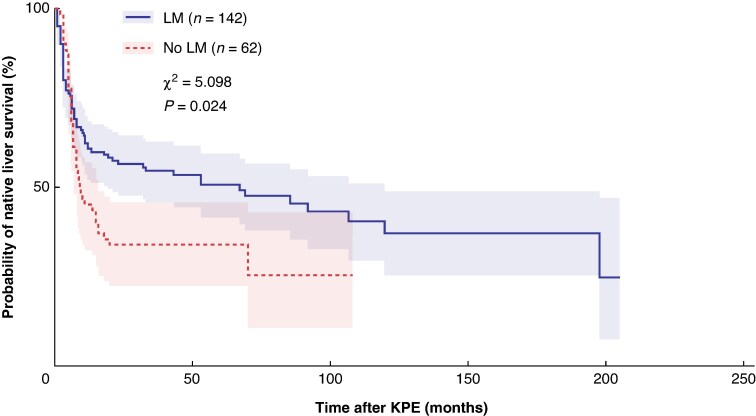
Kaplan–Meier curves for the probability of postoperative native liver survival in the LM and No LM groups Dashed lines represent 95% confidence intervals. LM, liver mobilization; KPE, Kasai portoenterostomy.

Neither the two- nor three-category outcome distribution at 6 months after KPE showed a significant difference between groups, but there was a trend towards higher rates of jaundice-free patients in the LM than No LM group (55% (78) *versus* 44% (27); *P* = 0.171; *Fig. 2* and *Fig. S2*). Native liver survival at the time of last follow-up was significantly higher in the LM group (*P* = 0.024; *Fig. 3*).

An exploratory correlation between annual centre caseload and 6-month jaundice clearance showed no significant association (Spearman *r* = −0.20, *P* = 0.722; *Fig. S1*).

### Liver transplantation

Patients without LM during KPE had significantly longer operative times (*P* < 0.001) and required greater intraoperative blood transfusion volumes (*P* < 0.001) compared to the LM group during liver transplantation (*Table 5*).

**Table 5:** Details of transplant surgery

	LM group		No LM group		P*
	Mean(s.d.)	*n*	Mean(s.d.)	*n*	
Age at LT (months)	18.3(33.6)	71	9.3(10.6)	42	< 0.001
Transfusion (ml/kg)	55.3(43.0)	55	95.81(61.22)	41	< 0.001
Surgery duration (min)	295.3(65.9)	55	509.8(167.5)	41	< 0.001

LM, liver mobilization s.d., standard deviation; LT, liver transplantation; min, minutes. *Unpaired t-test.

## Discussion

The findings of this study indicate that LM during KPE has short-term impacts, as reflected by the need for more intensive intraoperative anaesthetic management, postoperative changes in laboratory measurements, and signs of increased fluid retention, but was associated with significantly improved native liver survival without adverse effects on long-term outcomes or LT.

The No LM group was, on average, slightly younger, which may partly explain their lower bilirubin, higher albumin, and lower preoperative weight, consistent with a less advanced disease stage at the time of surgery. To date, no correlation between preoperative laboratory values and KPE outcome has been established^17^. Age at KPE remains the only widely accepted preoperative prognostic factor^18^, and it did not significantly differ between groups in the present study.

Haemodynamic instability during liver exteriorization in KPE can be effectively managed by experienced paediatric anaesthetists^8^, typically through intravenous fluid resuscitation and the use of catecholamines. Minor baseline differences between groups in pH and base excess at the start of surgery likely reflect variation in preoperative fluid management and anaesthetic practice between centres. Although such factors may have contributed to the higher intraoperative fluid requirements in the LM group, they are unlikely to fully explain the observed difference, which more plausibly reflected the haemodynamic impact of LM itself. Continuous intraoperative monitoring with near-infrared spectroscopy could provide additional insights into the cerebral perfusion effects of LM^19^.

After KPE, patients in the LM group exhibited more pronounced signs of stress (lactate levels and liver enzymes), likely reflecting intraoperative haemodynamic compromise and mechanical stress on the liver resulting from the mobilization procedure. LM patients more frequently required diuretics and had delayed abdominal drain removal, which may be attributable to increased hepatic stress and lymphatic leakage from the dissection of hepatic ligaments. These findings are consistent with those reported by Xu *et al*.^12^, who reported increased postoperative ascites and a longer hospital stay after KPE with LM and found no differences in liver enzyme levels between their study groups 2 weeks after KPE.

There was a trend towards a higher proportion of jaundice-free patients in the LM than No LM group 6 months after KPE, and long-term native liver survival was significantly improved in patients who underwent LM. These findings contrast with those reported by Park *et al*.^13^, who observed no significant differences in outcomes between their LM and No LM groups. Similarly, Xu *et al*.^12^ reported comparable rates of jaundice clearance at 3 months and slightly prolonged native liver survival in the No LM group at 1 and 2 years after KPE, but their long-term outcomes were not subjected to statistical analysis. Both studies are limited by their single-centre design and smaller sample sizes, which may preclude generalizable conclusions.

Early postoperative outcomes in this cohort showed similar, or even slightly higher, rates of LT in the LM group, which later reversed, resulting in overall superior native liver survival. LM may initially overwhelm a subset of vulnerable patients due to acute hepatic stress, leading to early deterioration and transplantation, but LM may also allow improved exposure and a more complete portal plate dissection, potentially resulting in more effective bile drainage and consequently more favourable long-term outcomes in other infants. It is conceivable that transient alterations in hepatic perfusion during mobilization could activate protective pathways with longer-term benefit, conceptually analogous to ischaemic conditioning^20,21^, although direct evidence for such mechanisms in this context is lacking and this theory remains highly speculative. Cholangitis is generally considered a negative prognostic factor for native liver survival in BA^22^, but the LM group in the present cohort nonetheless demonstrated superior long-term native liver survival. The reasons for this apparent discrepancy remain unclear and should be interpreted with caution given the retrospective nature of the data.

Patients in the No LM group required transplantation at a substantially younger age, often during the particularly vulnerable period of 1 year of age^23–25^, which reflects their less favourable native liver survival. LM appeared to prolong native liver survival sufficiently to delay transplantation into a more favourable age range, potentially facilitating technically less challenging procedures and aligning with the primary therapeutic goals of KPE. Previous studies suggest that key LT outcome parameters (for example, graft survival) are not affected by previous KPE (regardless of LM), although higher rates of intraoperative bleeding, bowel perforations, and biliary complications have been reported^26^.

The main limitations of this study were its retrospective design and the inherent heterogeneity between participating centres, which differed in patient volume, referral patterns, case mix, and perioperative management. The distribution of patients undergoing LT *versus* retaining their native liver varied across centres, and the long study period of up to 17 years introduced further variability, because surgical techniques and postoperative management protocols may have evolved over time. The availability and granularity of data related to intraoperative and perioperative management, and especially transplant-specific parameters, were inconsistently documented across centres, precluding definitive conclusions regarding the impact of previous LM on subsequent LT because differences in operative complexity cannot be excluded. Because the decision to perform LM was made at the centre level, LM status was largely collinear with centre, precluding meaningful adjustment for centre effects. The crossing of the Kaplan–Meier curves warrants careful interpretation and should be regarded as hypothesis-generating rather than confirmatory.

Future prospective multicentre studies with standardized data collection, predefined surgical and perioperative variables, and systematic documentation of transplant-related outcomes will be required to more definitively assess the impact of LM during KPE.

LM during KPE was associated with increased perioperative hepatic stress but favourable long-term outcomes in this cohort, without evidence of adverse effects on subsequent LT. Prospective standardized multicentre studies are warranted to further clarify the potential benefits and risks of LM and to determine whether these findings are generalizable across surgical settings.

## Supplementary Material

zrag064_Supplementary_Data

## Data Availability

All relevant aggregated data are included in the article. The underlying retrospective patient-level data are not publicly available due to privacy and ethical restrictions but may be made available by the corresponding author upon reasonable request and appropriate approvals.
