# Supplementary material for: Liver mobilization during Kasai portoenterostomy: retrospective multicentre analysis
Source: BJS Open. 2026 Jun 8;10(3):zrag064. doi: 10.1093/bjsopen/zrag064 (PMC13245721; doi:10.1093/bjsopen/zrag064)
Supplement: zrag064_Supplementary_Data [file zrag064_supplementary_data.docx]

**Liver mobilisation during Kasai portoenterostomy: retrospective multicentre analysis**

Marie Uecker^1,2^, Maximilian Holweg^3^, Maria Hukkinen^4^, Lucas Moratilla-Lapeña^5^, Kristine Dräger^2^, Alexander Domasch^1^, Katja Nickel^6^, Sören Wiesner^7^, Cornelius Jakob van Beekum^8^, Jörg Fuchs^3^, Uta Herden^9^, Francisco Hernández^5^, Mikko Pakarinen^4^, Riccardo Superina^10^, Joachim F. Kuebler^11^, Christoph Slavetinsky*^3^, Omid Madadi-Sanjani*^2,9^

^1^ Department of Pediatric Surgery, Hannover Medical School, Hannover, Germany.

^2^ Department of Pediatric Surgery, University Medical Center Hamburg-Eppendorf, Hamburg, Germany.

^3^ Department of Pediatric Surgery and Urology, University Children's Hospital Tübingen, Tübingen, Germany.

^4^ Section of Pediatric Surgery, New Children's Hospital, University of Helsinki, Finland

^5^ Department of Paediatric Surgery, La Paz Children's University Hospital, Madrid, Spain.

^6^ Clinic of Anesthesiology and Intensive Care Medicine, Hannover Medical School, Hannover, Germany

^7^ Institute of Biostatistics, Hannover Medical School, Germany.

^8^ Department of General, Visceral, & Transplant Surgery, Transplant Center Hannover, Hannover Medical School, Hannover, Germany.

^9^ Department of Visceral Transplantation, University Medical Center Hamburg-Eppendorf, Hamburg, Germany

^10^ Division of Transplant and Advanced Hepatobiliary Surgery, Ann and Robert H Lurie Children's Hospital of Chicago, Chicago, Illinois, USA

^11^ Department of Pediatric Surgery and Urology, Hospital Bremen-Mitte, Bremen, Germany

*These authors contributed equally to this work

**Corresponding author:**

Marie Uecker, MD

Department of Pediatric Surgery

University Medical Center Hamburg-Eppendorf

Martinistr. 52

20251 Hamburg, Germany

Mail: m.uecker@uke.de

Tel: +49-152-22806700

Fax: +49-40-74104060

**ORCID-ID: 0009-0007-2582-2368**

**Supplementary Materials - Index**

| **Supplementary Figures and Tables** |  |
| --- | --- |
| Supplementary Figure 1 | *pag. 3* |
| Supplementary Figure 2 | *pag. 3* |
|  |  |

**Supplementary Figures**

**
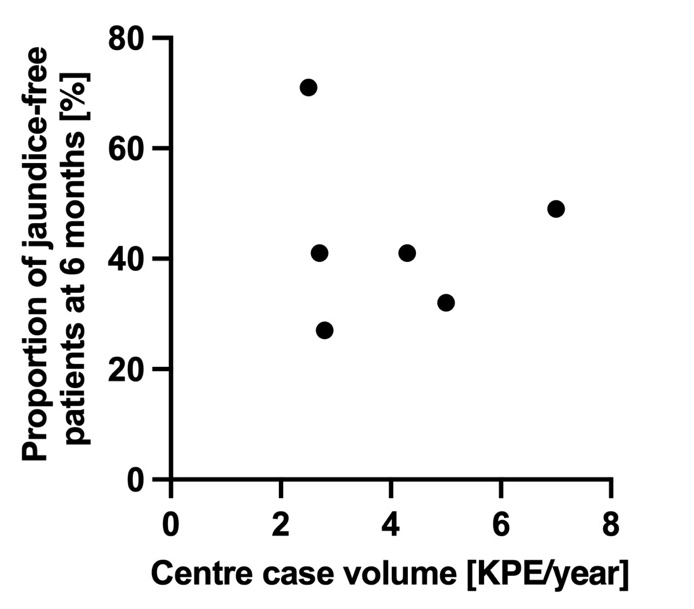
**

**Suppl. Fig. 1:** Correlation between centre case volume and 6-months jaundice clearance after Kasai portoenterostomy (KPE).
Scatter plot showing the relationship between annual centre KPE volume and the proportion of patients achieving jaundice clearance 6 months postoperatively. Each dot represents one participating centre. No significant correlation was observed between centre case volume and outcome (Spearman r = –0.2, p = 0.72).


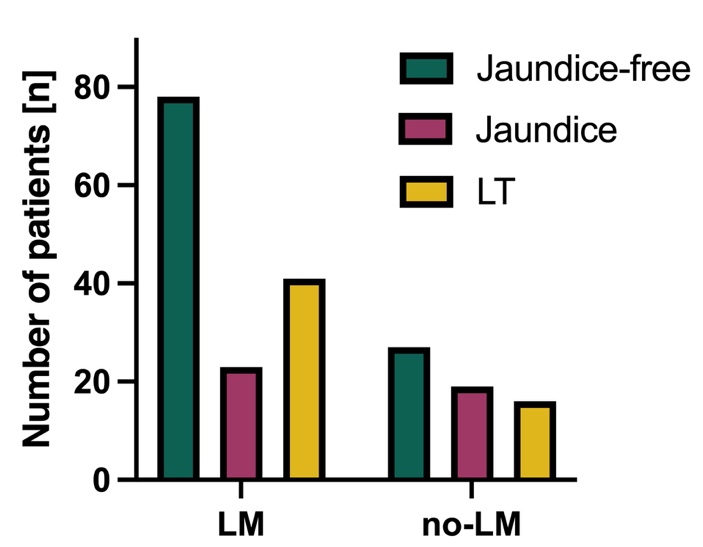


**Suppl. Fig. 2:** Clinical outcome at six months post-KPE presented as three separate categories (jaundice-free, jaundice, LT) for each group. LM group (n=142): jaundice-free n=78 (54.9%), jaundice n=23 (16.2%), LT n=41 (28.9%). No-LM group (n=62): jaundice-free n=27 (43.5%), jaundice n=19 (30.6%), LT n=16 (25.8%). Group differences were analysed by Fisher’s exact test (*p*=0.66).
